# Behavioral and psychosocial factors and their effects on insomnia among people undergoing entry quarantine in hotels during COVID-19 pandemic: a cross-sectional study in Guangzhou, China

**DOI:** 10.1186/s12889-023-15340-4

**Published:** 2023-05-30

**Authors:** Rui Luo, Jinghua Li, Menglin Shang, Zhili Peng, Zhiwei Wang, Jing Gu

**Affiliations:** 1 Department of Medical Statistics, School of Public Health, Sun Yat-Sen University, No.74, Zhongshan Second Road, Guangzhou, 510080 China; 2 Sun Yat-Sen University Global Health Institute, School of Public Health and Institute of State Governance, Sun Yat-Sen University, Guangzhou, 510080 China; 3 Guangdong Key Laboratory of Health Informatics, Guangzhou, 510080 China; 4 Department of Logistics and Security, Guangzhou Center for Disease Control and Prevention, Guangzhou, China; 5 Department of 12320 Health Hotline, Guangzhou Center for Disease Control and Prevention, Guangzhou, China; 6 Guangzhou Joint Research Center for Disease Surveillance, Early Warning, and Risk Assessment, Guangzhou, China

**Keywords:** Insomnia, Covid-19, Mental health, Behavioral health, Quarantined population

## Abstract

**Background:**

The COVID-19 pandemic continues to impact global health and China requires a 14-day quarantine for individuals on flights with positive COVID-19 cases. This quarantine can impact mental well-being, including sleep. This study aims to examine the impact of psychosocial and behavioral factors on insomnia among individuals undergoing quarantine in hotels.

**Methods:**

This study was a cross-sectional survey carried out in Guangzhou, China. The data was gathered through online questionnaires distributed to international passengers who arrived in Guangzhou on flights and were required to undergo a 14-day quarantine in hotels arranged by the local government. The questionnaires were sent to the participants through the government health hotline "12,320."

**Results:**

Of the 1003 passengers who were quarantined, 6.7% reported significant anxiety and 25.0% had varying degrees of insomnia. Anxiety was positively associated with insomnia (β = 0.92, *P* < 0.001), while collectivism (β = -0.07, *P* = 0.036), indoor exercise (β = -0.50, *P* < 0.001), and the perceived people orientation of the public health service (β = -0.20, *P* = 0.001) were negatively associated with insomnia. The study also identified moderating effects, such that a higher sense of collectivism, a greater frequency of indoor exercise, and a higher perception of the people-oriented of the public health service were associated with a lower impact of anxiety on insomnia. These moderating effects were also observed in participants with varying degrees of insomnia.

**Conclusions:**

This study reveals that a proportion of people undergoing entry quarantine experience insomnia and confirms how psychosocial and behavioral factors can alleviate insomnia in this population.

**Supplementary Information:**

The online version contains supplementary material available at 10.1186/s12889-023-15340-4.

## Background

The ongoing coronavirus pandemic (COVID-19) that emerged in early 2020 continues to pose a significant threat to human life and well-being due to the rapid spread and highly infectious nature. It presents an unparalleled challenge to the global public health system and governments worldwide [1]. In 2021, the main source of COVID-19 cases in China was from imported cases from other countries. Despite the pandemic's widespread presence globally, the virus continues to evolve, with the latest Omicron strain being highly contagious, making it even harder to control and prevent imported cases [2].

During this global pandemic, the strategies employed by different countries vary based on the government and individual circumstances in each nation. Mainland China currently requires that all individuals arriving on a flight with COVID-19 positive passengers must undergo a 14-day quarantine at a designated COVID-19 quarantine hotel. This centralized quarantine is a public health measure that restricts the mobility of affected individuals in order to safeguard public health [3]. However, the quarantine can have varying impacts on the mental well-being of those in isolation due to the threat of infection and the 14-day period of observation within a confined quarantine setting [4].

The literatures have primarily focused on depression and anxiety among populations undergoing quarantine. however, there have been few studies that have specifically investigated the impact of quarantine on sleep [4]. Quality of sleep is crucial during the COVID-19 pandemic. Firstly, sleep plays a crucial role in regulating mood and any sleep disorders can have a direct impact on mood regulation the following day [5]. Additionally, insufficient sleep can weaken the immune system, making individuals more susceptible to viral infections [6]. Furthermore, during the 14-day quarantine period, many people may be required to work remotely, and the quality of sleep on a given night has a significant impact on the quality of work output the following day. Therefore, it is important to gain an understanding of the quality of sleep among individuals undergoing entry quarantine in hotels.

Among the factors that can impact an individual’s sleep, anxiety has been strongly associated with insomnia [7]. Approximately 75% of individuals with Generalized Anxiety Disorder (GAD) experience insomnia [8], and the severity of insomnia has been shown to be directly proportional to the severity of anxiety [9]. Studies from China have shown that individuals undergoing quarantine have significantly higher rates of anxiety compared to those who are not in quarantine [10, 11]. However, these studies were limited to residents in China and did not investigate insomnia and anxiety among individuals undergoing entry quarantine. Individuals undergoing entry quarantine come from countries with varying pandemic prevention policy, some of which may be less strict, and it is crucial to take into consideration their experiences with insomnia and anxiety in this rapidly changing environment.

During the COVID-19 pandemic, China has implemented policies to maintain social distancing and centralized quarantine, which were supported by a range of public services and community involvement. The concept of collectivism in China emphasizes the importance of acting in the best interest of the community [12]. Individuals with a stronger sense of collectivism are more willing to comply with health regulations [12]. Social responsibility is a reliable predictor of various positive psychosocial conditions, including adherence to social distancing measures,, which can take many forms [13]. In times of health crisis, individuals with a strong sense of social responsibility are less likely to experience anxiety [14]. In addition, community-based public health services play an important role in China’s efforts to prevent and control the spread of the epidemic. These services, which prioritize the needs of the people, offer comfort and reassurance to those in quarantine and can help alleviate their insomnia. However, the relationship between collectivism, social responsibility, and perceptions of public health services and the insomnia of individuals undergoing entry quarantine is yet to be thoroughly explored.

Behavioral factors can influence an individual’s mental health. Engaging in physical exercise can significantly reduce mental health problems, such as anxiety, that may arise from public crises [15, 16]. It is recommended that individuals engage in appropriate physical exercise to improve their mental health during the COVID-19 pandemic [17]. In addition, playing appropriate online games has been shown to have a positive impact on an individual's mental health [18]. Communicating with friends and watching online videos can also have a positive effect on mental health during COVID-19 pandemic [19, 20]. It is expected that these behaviors also may help to improve the sleep of individuals undergoing entry quarantine in hotels.

The objectives of this study were to investigate the association between insomnia and anxiety among individuals undergoing entry quarantine in hotels during the COVID-19 pandemic, and to explore the moderating effects of psychosocial factors (e.g., collectivism and perceived people orientation of the public health service) and behavioral factors (e.g., online communication and indoor exercise) on insomnia.

## Methods

### Study design

This cross-sectional study was conducted in Guangzhou, the largest city in southern China, with a population of 15 million. Guangzhou Baiyun International Airport is the largest transportation hub in mainland China, with a high volume of passengers. All passengers arriving in Guangzhou from international flights are required to undergo a 14-day quarantine in hotels organized by the local government. The data for this study was collected from August 9 to August 16, 2021, through an online questionnaire that was distributed via the government health hotline “12,320” to all incoming passengers who met the inclusion criteria.

### Participants

The participants in the study, individuals had to meet the following inclusion criteria: (a) passengers arriving in Guangzhou on an international flight with confirmed positive cases of COVID-19; (b) passengers currently undergoing 14 days of quarantine observation in a quarantine hotel; (c) those with mobile communication devices such as cell phones or tablets who could independently complete various scales. Informed consent was obtained from all individual participants included in the study. During the survey period, a total of 1,520 people met the inclusion criteria, of which 1,259 completed the questionnaire. Questionnaires that did not answer the quality control questions correctly were excluded, resulting in a final sample size of 1,003.

### Study process

During the study, the questionnaire was designed using the online survey tool Questionnaire Star (www.wjx.cn) and was distributed to eligible participants through the Guangzhou Health Hotline “12,320” platform. The Guangzhou 12,320 Health Hotline is a credible and authoritative communication window for the entire medical and health industry in Guangzhou, a department under the Guangzhou Center for Disease Control and Prevention, led by the Guangzhou municipal health Commission. The online questionnaire link was distributed by 12,320 Health Hotline staff through a mobile phone text message. All the participants were informed of the study background and anonymity, and once the questionnaire was submitted, consent was implied.

### Measurements

#### Sociodemographic characteristics

The questionnaire collected the sociodemographic characteristics of the participants, including gender, age, education, marital status, monthly income, job, and nationality.

#### Entry-related information

Entry-related information included the reason for entry, length of the last stay abroad and COVID-19 vaccination status.

#### Insomnia

Insomnia was assessed using the Insomnia Severity Index [21]. This scale comprises seven items and has a maximum score of 28: 0–7 for no insomnia, 8–14 for subclinical insomnia, 15–21 for moderate insomnia, and 22–28 for severe insomnia [22]. The scale was used to assess the severity of insomnia during quarantine period. Higher scores indicate more severe insomnia. The scale showed good reliability and validity in this study, with a Cronbach’s alpha of 0.90.

#### Anxiety

Anxiety during quarantine period was assessed using the Chinese version of the Generalized Anxiety Disorder Scale (GAD-7) [23]. The scale consists of seven items and uses 4-ponit Likert scales. The score range is 0–21. The higher the score, the more severe the anxiety, with 10 generally considered the cutoff point for significant anxiety. The Chinese version of the GAD-7 has been shown to have good reliability and validity [24], and Cronbach’s alpha was 0.95 in this study.

#### Perceived people orientation of the public health service

The perceived people orientation of the public health service is a measure developed by Bolin Cao et al. [25]. To assess the understanding, care, and credibility of the public health officials and staff assigned to support the daily lives of people undergoing entry quarantine. It includes three items, “Public health service workers answered my questions in a way that I could understand,” “Public health service workers cared about my feelings and emotions,” and “I thought the public health service workers were trustworthy” [25]. Responses were rated on a 5-point Likert scale (1 = strongly disagree to 5 = strongly agree). The higher the score, the higher the perceived people orientation of the public health service. The scale showed good reliability with a Cronbach’s alpha of 0.95.

#### Collectivism

Collectivism was developed by the research group based on a study by Wagner and Moher [26]. Responses were measured by six items (e.g., “Individuals should sacrifice their interests for the collective”) on a 5-point Likert scale (1 = strongly disagree to 5 = strongly agree). Higher scores indicated greater sense of collectivism. Cronbach’s alpha was 0.92, showing that the scale had good reliability.

#### Social responsibility

Social responsibility was developed by the research team according to the Anderson-Butcher D et al. [27]. It was measured by three items ranked on a 5-point Likert scale (1 = strongly disagree to 5 = strongly agree). The items were “Everyone should spend some time making his city or country better,” “It is everyone’s duty to do their best to get the job done,” and “You feel bad when you can’t do the work you promised you would do.” Higher scores indicated stronger social responsibility. The scale showed good reliability with a Cronbach’s alpha of 0.79.

#### Behavior during quarantine period

Four types of behavior during the period of quarantine were studied, including online communication, playing online games, watching online videos, and doing indoor exercise. The questions were specifically designed for this study, and each was answered on a 5-point Likert scale (1 = never to 5 = always), with higher scores indicating a higher frequency of the behavior during quarantine period.

### Statistical analysis

Continuous variables were described by mean ± standard deviation (M ± SD), and categorical variables were described by frequency (percentage). A multiple linear regression model was used with insomnia as the dependent variable and anxiety, psychosocial and behavioral variables (i.e., collectivism, indoor exercise) as independent variables. The model was adjusted for potential confounding factors, namely gender, age, education, marital status, job, monthly income, nationality, reason for entry, length of the last stay abroad and COVID-19 vaccination status. To assess the moderating effects of psychosocial and behavioral factors on the association between anxiety and insomnia, the independent variables that were significantly related to insomnia in the previous multiple linear regression analysis were used as candidate moderators. According to the moderating effect detection rules, we used the hierarchical multiple regression approach [28]. The variables were included in this analysis in three steps: first, mean-centering was carried out for the anxiety and candidate moderators. Second, we included the variables that were significantly related to insomnia and anxiety. Third, we added the interaction terms between the variables significantly related to insomnia and anxiety, such as indoor exercise × anxiety. If the interaction terms were significant (*P* < 0.05), we conducted a simple slopes test to examine the interaction effect at high and low levels (i.e., one standard deviation above and below the mean, respectively). We also did a subgroup analysis of participants with varying degrees of insomnia. The moderating effects were explained by the figure based on the slopes test. *P* < 0.05 was considered statistically significant with* α* = 0.05, using a two-tailed test. SPSS 26.0 (SPSS, Inc., Chicago, IL, USA) and R 4.2.1 were used for our statistical analysis.

## Results

### Background information of participants

Among the 1003 participants, 828 (82.6%) were men, the mean age was 36.9 years (SD = 10.2), and 33.4% had a bachelor’s degree or above. The majority of the participants (63.3%) were married, approximately half (40.1%) worked as company staff, and about half (44.9%) earned between 5000 and 12,000 yuan (US$ 751 and US$ 1803) per month. Most of the participants (98.4%) were Chinese. In terms of reason for entry, 26.3% of the participants were arriving to live in mainland China, and approximately half (44.3%) had been abroad for more than 1 year. More than half (62.4%) of the participants were fully vaccinated with COVID-19 vaccine. The descriptive statistics of the participants are presented in Table 1.

**Table 1:** Background information of participants (*N* = 1003)

Variable	*N* (%) / M ± SD
Gender	
Male	828 (82.6)
Female	175 (17.4)
Age	36.9 ± 10.2
Education	
Junior high and below	278 (27.7)
High school	228 (22.7)
Junior college	162 (16.2)
Bachelor’s degree and above	335 (33.4)
Marital status	
Single	317 (31.6)
Married	635 (63.3)
Divorced and other	51 (5.1)
Job	
Company staff	402 (40.1)
Workers	199 (19.8)
Students	38 (3.8)
Other (e.g., government staff)	364 (36.3)
Monthly income	
No income	195 (19.4)
< 5000 yuan (US$ 751)	137 (13.7)
$$\ge$$5000 yuan	450 (44.9)
$$\ge$$ 12,000 yuan (US$ 1803)	221 (22.0)
Nationality	
Chinese	987 (98.4)
Non-Chinese	16 (1.6)
Reasons for entry	
Live	264 (26.4)
Visiting family	175 (17.4)
Work	176 (17.5)
Avoiding foreign pandemics	208 (20.8)
Other	180 (17.9)
Length of last stay abroad	
Within a month	95 (9.5)
1 to 3 months	102 (10.1)
4 to 6 months	134 (13.4)
7 to 12 months	228 (22.7)
More than 1 year	444 (44.3)
COVID-19 vaccination status	
fully vaccinated	626 (62.4)
vaccinated with one dose	27 (2.7)
Not vaccinated	350 (34.9)

### Psychosocial and behavioral information

There were 251 people (25%) with varying degrees of insomnia, and 23.1% of them had significant anxiety. The means and standard deviations of the psychosocial scales were as follows: collectivism, mean = 23.6 (SD = 4.5); social responsibility, mean = 12.7 (SD = 1.7), and perceived people orientation of the public health service, mean = 12.5 (SD = 2.4). The detailed psychosocial scale items were in supplementary table 1. In terms of behavioral factors, the majority (86.3%) of participants often or always online communicated with people. Half (51.9 %) of the participants often or always played online games. About 70.0% of participants of often or always did indoor exercise or watched online videos. The full results are reported in Table 2.

**Table 2:** Psychosocial status and behavior during centralized quarantine (N (%) / M ± SD)

Variable	Total group (*N* = 1003)	Insomnia subgroup (*N* = 251)
Insomnia	5.0 ± 5.7	13.4 ± 4.9
No insomnia	752 (75.0)	NA
Subclinical insomnia	169 (16.8)	169 (67.3)
Moderate insomnia	63 (6.3)	63 (25.1)
Severe insomnia	19 (1.9)	19 (7.6)
Anxiety	2.6 ± 4.0	6.7 ± 4.9
Significant anxiety	67 (6.7)	58 (23.1)
Nonsignificant anxiety	936 (93.3)	193 (76.9)
Psychosocial status		
Collectivism	23.6 ± 4.5	23.1 ± 4.5
Social responsibility	12.7 ± 1.7	12.5 ± 1.7
Perceived people orientation of public health service	12.6 ± 2.4	12.1 ± 2.4
Behavioral status		
Playing online games		
Never and rarely	353 (35.2)	100 (39.8)
Sometimes	129 (12.9)	29 (11.6)
Often and always	521 (51.9)	122 (48.6)
Indoor exercise		
Never and rarely	121 (12.1)	37 (14.8)
Sometimes	160 (16.0)	53 (21.1)
Often and always	722 (71.9)	161 (64.1)
Watching online videos		
Never and rarely	128 (12.7)	32 (12.7)
Sometimes	164 (16.3)	51 (20.3)
Often and always	712 (71.0)	168 (70.0)
Online communication		
Never and rarely	63 (6.3)	11 (4.4)
Sometimes	74 (7.4)	21 (8.4)
Often and always	866 (86.3)	219 (87.2)

### Factors associated with insomnia

The results of the multiple linear regression analysis showed that after adjusting for confounding factors, there was a significant correlation between anxiety and insomnia, with a corresponding increase in insomnia as anxiety increased (*β* = 0.92, 95%*CI* = 0.85, 0.98, *P* < 0.001). Insomnia also showed a significant negative correlation with perceived people orientation of the public health service and collectivism, such that higher perceived people orientation of the public health service (*β* = -0.20, 95%*CI* = -0.28, -0.06, *P* = 0.001) and a greater sense of collectivism were associated with milder insomnia (*β* = -0.07, 95%*CI* = -0.14, -0.01, *P* = 0.036). In addition, the higher frequency of indoor exercise, the milder the insomnia (*β* = -0.50, 95%*CI* = -0.77, -0.24, *P* < 0.001). The details are shown in Table 3.

**Table 3:** Multiple linear regression analysis of insomnia* (*N* = 1003)

	*β*	SD	95% CI	*P*
Anxiety	0.92	0.03	0.85, 0.98	< 0.001
Psychosocial characteristics				
Perceived people orientation of public health service	-0.20	0.06	-0.28, -0.06	0.001
Social responsibility	0.14	0.09	-0.04, 0.31	0.131
Collectivism	-0.07	0.04	-0.14, -0.01	0.036
Behavior during centralized quarantine				
Online communication	0.13	0.16	-0.19, 0.45	0.418
Online gaming	-0.05	0.11	-0.26, 0.15	0.609
Watching online videos	0.19	0.15	-0.10, 0.48	0.206
Indoor exercise	-0.50	0.13	-0.77, -0.24	< 0.001

*The model was adjusted for gender, age, education, marital status, job, monthly income, nationality, reason for entry, length of last stay abroad and COVID-19 vaccination status

### Insomnia-related moderating effects

**Fig. 1:**
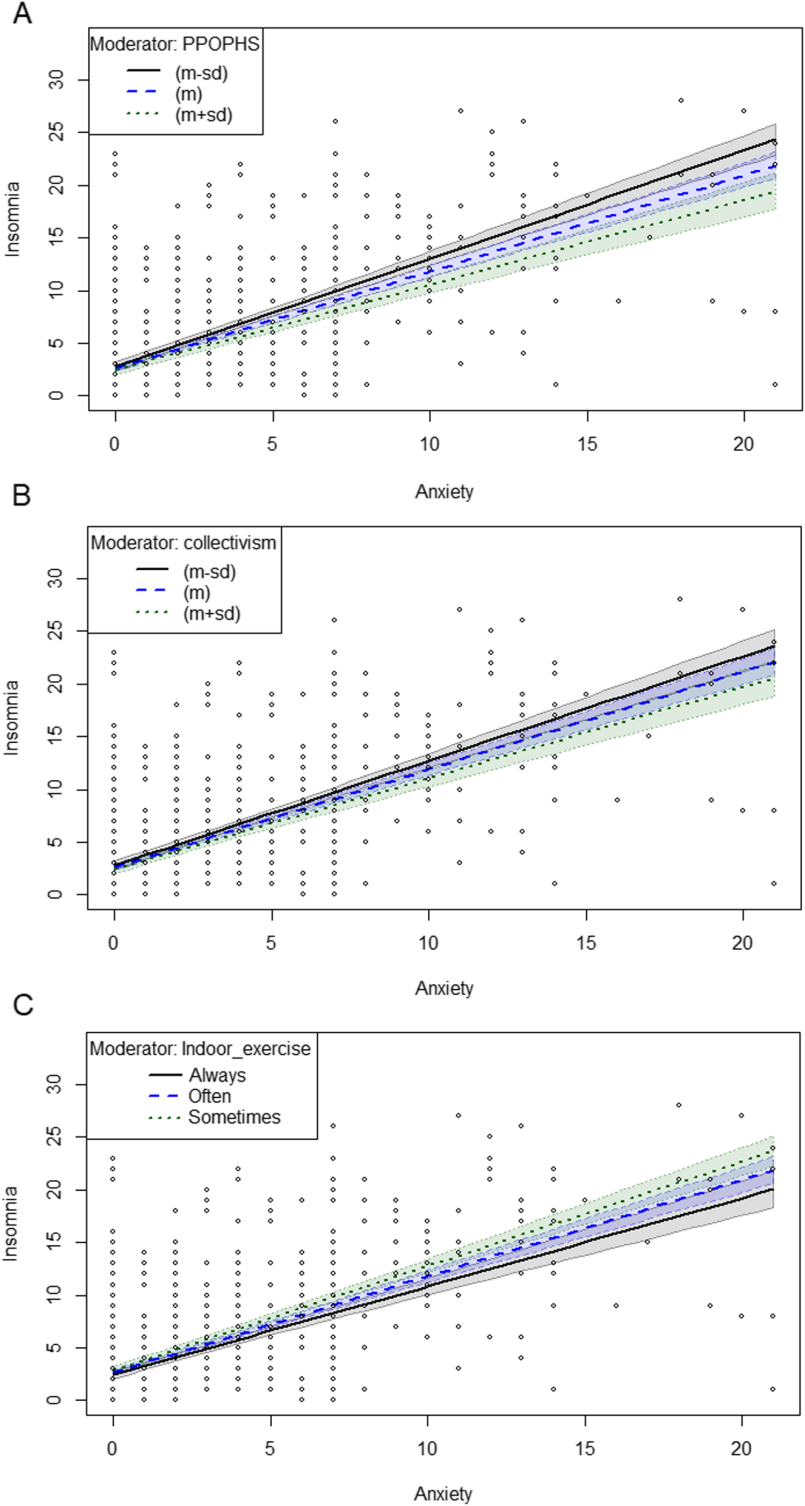
Moderation of the effect of anxiety on insomnia. PPOPHS: Perceived people orientation of the public health service

**Fig. 2:**
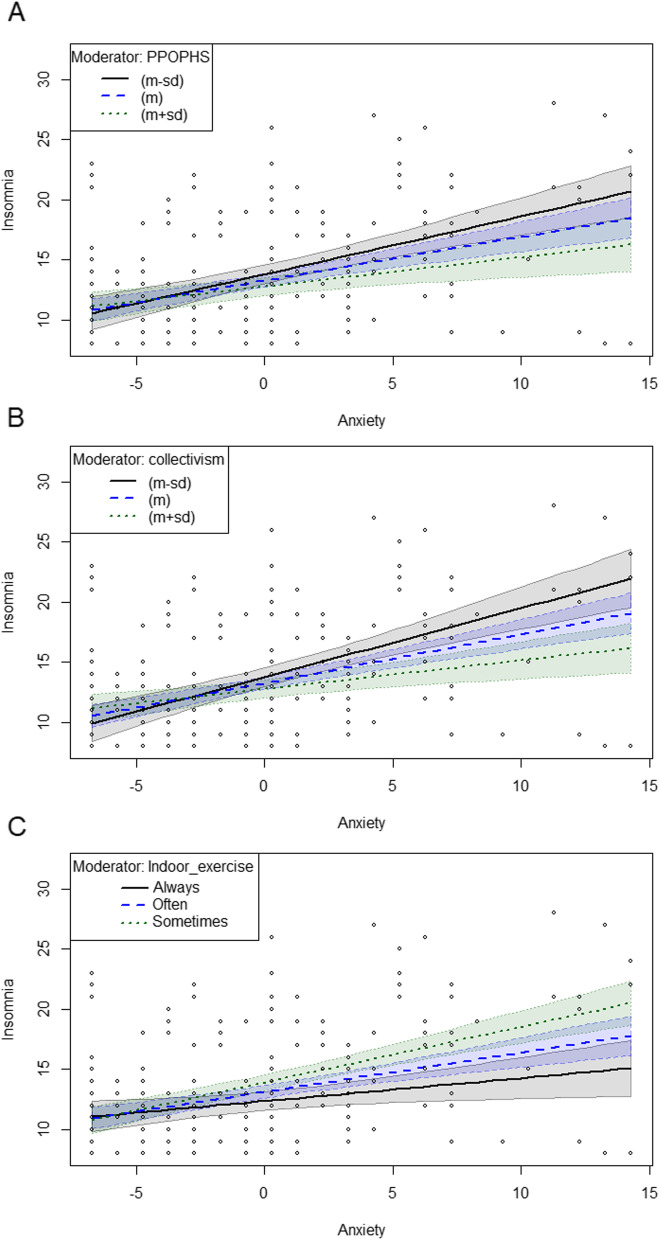
Moderation of the effect of anxiety on insomnia in subgroup. PPOPHS: Perceived people orientation of the public health service

In the previous multiple linear regression analysis, we selected the variables that had statistically significant associations with insomnia (collectivism, perceived people orientation of the public health service, and indoor exercise) and used them as candidate moderators to analyze their moderating effects between anxiety and insomnia, respectively. Table 4 showed that collectivism moderated the relationship between anxiety and insomnia (*β* = -0.02, *P* = 0.025). A similar moderating effect was shown for perceived people orientation of the public health service (*β* = -0.05, *P* < 0.001) and the frequency of indoor exercise (*β* = -0.08, *P* = 0.004), as shown in Fig. 1. The same moderating effects were found in subgroup analyses of participants with insomnia, as shown in Table 5 and Fig. 2.

**Table 4:** Moderating effects of anxiety on insomnia* (*N* = 1003)

Model	Step	Variable	*β*	*P*	*Δ* ${R}^{2}$	*ΔF*	*P*
1	1	Anxiety	0.93	< 0.001	0.47	74.06	< 0.001
		Collectivism	-0.08	0.008			
	2	Anxiety	1.27	< 0.001	0.01	5.01	0.025
		Collectivism	-0.04	0.276			
		Anxiety* collectivism	-0.02	0.025			
2	1	Anxiety	0.93	< 0.001	0.48	75.39	< 0.001
		Perceived people orientation of public health service	-0.22	< 0.001			
	2	Anxiety	1.48	< 0.001	0.01	13.95	0.011
		Perceived people orientation of public health service	-0.08	0.222			
		Anxiety* perceived people orientation of public health service	-0.05	< 0.001			
3	1	Anxiety	0.93	< 0.001	0.48	74.99	< 0.001
		Indoor exercise	-0.42	< 0.001			
	2	Anxiety	1.22	< 0.001	0.01	8.24	0.004
		Indoor exercise	-0.22	0.109			
		Anxiety* indoor exercise	-0.08	0.004			

*The models were adjusted for gender, age, education, marital status, job, monthly income, nationality, reason for entry, length of last stay abroad and COVID-19 vaccination status

**Table 5:** Moderating effects of anxiety on insomnia in the subgroup* (*N* = 251)

Model	Step	Variable	*β*	*P*	*Δ* ${R}^{2}$	*ΔF*	*P*
1	1	Anxiety	0.36	< 0.001	0.26	7.06	< 0.001
		Collectivism	-0.14	0.025			
	2	Anxiety	1.26	< 0.001	0.03	10.18	0.002
		Collectivism	0.14	0.192			
		Anxiety* collectivism	-0.04	0.002			
2	1	Anxiety	0.36	< 0.001	0.22	17.815	< 0.001
		Perceived people orientation of public health service	-0.25	0.030			
	2	Anxiety	0.95	< 0.001	0.02	5.66	0.018
		Perceived people orientation of public health service	0.12	0.534			
		Anxiety* perceived people orientation of public health service	-0.05	0.018			
3	1	Anxiety	0.36	< 0.001	0.28	7.86	< 0.001
		Indoor exercise	-0.84	0.001			
	2	Anxiety	0.87	< 0.001	0.03	9.79	0.002
		Indoor exercise	0.14	0.720			
		Anxiety* indoor exercise	-0.14	0.002			

*The models were adjusted for gender, age, education, marital status, job, monthly income, nationality, reason for entry, length of last stay abroad and COVID-19 vaccination status

## Discussion

This study established a positive association between anxiety and insomnia among 1003 individuals undergoing entry quarantine in hotels. Our findings indicated that collectivism, the frequency of indoor exercise, and perceived people orientation of the public health service could moderate the impact of anxiety on insomnia. Participants who had a greater sense of collectivism, experienced higher quality people-oriented public health services, and engaged in more frequent indoor exercise were found to have a weaker effect of anxiety on their insomnia during the quarantine period.

The implementation of centralized quarantine during the COVID-19 pandemic has brought to light numerous mental health problems, including anxiety, among the general population [29]. Sleep, being a physiological process that is greatly impacted by the environment, can vary in response to changes in anxiety levels [29]. Our study results were in line with previous studies, as we found a significant positive association between anxiety and insomnia. As anxiety levels rose, we observed a corresponding increase in insomnia [30, 31]. Individuals with elevated levels of anxiety are prone to excessive worrying, a tendency to view ambiguous information in a negative light, and report lower sleep quality [32]. The results of this study highlight the importance of monitoring the sleep quality of individuals undergoing quarantine, as a way to minimize psychological harm and prevent a vicious cycle of anxiety-insomnia-anxiety. By doing so, the negative mental health impact of quarantine on the general public can be reduced.

Our study found that a stronger sense of collectivism was associated with a weaker impact of anxiety on insomnia. This has potential implications for public health interventions such as centralized quarantine during the COVID-19 pandemic. Individuals with a stronger sense of collectivism are more likely to act in the best interest of the community and are more willing to prevent harm [33]. Therefore, people are more likely to comprehend and accept the implementation of centralized quarantine and to maintain a relatively calm state of mind during this procedure. Collectivism was significantly associated with insomnia, but the effect size was not large (β = -0.07). The small effect sizes may indicate complex and multifaceted relationships between the predictor and outcome. For behaviors such as sleep, small improvements in promoting a collective mindset may have positive impacts on sleep quality at the population level [34]. Future research could explore the mechanisms through which collectivism impacts sleep quality.

Our study found that the higher quality people-oriented public health service corresponds to a weaker impact of anxiety on insomnia, emphasizing the significance of public health services in supporting individuals during quarantine in the COVID-19 pandemic [35, 36]. The primary services offered by public health organizations include monitoring of body temperature provision of daily meals and essential supplies, as well as guidelines for seeking medical assistance when required. According to the theory of person-centered care, public health workers are encouraged to exhibit empathy, respect, involvement, and individualized attention [37]. Despite the potential for public health services to become overwhelmed during an epidemic, maintaining a high-quality and people-oriented public health service can effectively mitigate public panic and increase trust in the government. Anxiety decreases when individuals feel understood and cared for by others, especially when receiving public health services from local governments and health organizations [38]. A higher quality people-oriented public health service can alleviate the psychosocial problems caused by negative media coverage and strengthen personal coping strategies to reduce negative emotions during the fight against a disease outbreak [39]. These services are typically delivered by front-line health workers in the community. Therefore, public health emergency preparedness should include training focused on addressing both public mental health and disease prevention, with the aim of improving the people-oriented approach of public health services in the future.

Our study found that engaging in indoor exercise during the quarantine period could reduce the impact of anxiety on insomnia. Studies have shown that regular physical exercise can be an effective non-pharmacological treatment for individuals with insomnia [40, 41]. Physical exercise can enhance sleep quality by modulating various body systems (such as the autonomic nervous system), stimulating the release of melatonin, and increasing energy expenditure and demand for sleep [42–45]. To improve sleep quality of individuals undergoing quarantine, clear guidelines for indoor exercise and provision of simple exercise equipment could be provided in quarantine hotels. As our study utilized exercise as a continuous variable and did not categorize the level of exercise, future studies exploring the association between varying amounts of indoor exercise and insomnia is necessary.

Quarantine, either in hotels or at home, was implemented as a measure to prevent the spread of COVID-19 [3]. During this time, the risk of infection, limited activities, and uncertainty can take a toll on the mental health of those who are quarantined [4]. To provide support to those in quarantine, different countries have recommended different approaches. For example, a study from Bulgaria supports that exposure to nature can have a positive impact on mental health during quarantine [46]. Another study from Hong Kong suggests that indirect contact with nature can be a protective factor for mental health during quarantine [47]. This study has several limitations. Firstly, data was not collected on pre-existing insomnia or anxiety, making it unclear if the participants' symptoms were caused by quarantine or pre-existing conditions. Secondly, we did not consider the quarantining time before the participants participated in the survey. The time might be a potential confounder as it could be related to insomnia or anxiety. Thirdly, due to the use of convenience sampling and the survey being conducted only among individuals undergoing entry quarantine in Guangzhou, the results may not be easily generalizable to other populations. Fourthly, although collectivism was found to have significant associations with insomnia, the effect size was small. Finally, the questionnaire was self-administered online, which raises concerns about the quality of the data collected. However, efforts were made to ensure data quality by setting quality control questions and relying on the official Guangzhou 12,320 Health Hotline platform for questionnaire distribution.

## Conclusions

In conclusion, this paper highlighted that a quarter of individuals undergoing entry quarantine experienced insomnia and established the association between anxiety and insomnia. Psychosocial and behavioral factors, such as the stronger sense of collectivism, more frequent indoor exercise, and higher quality people-oriented public health service, can alleviate the impact of anxiety on insomnia. Importantly, these findings provide evidence for the development of future guidelines related to COVID-19 and other public health scenarios that require personnel quarantine.

## Supplementary Information

**Additional file 1: Stable 1.** Specific items of psychosocial scales (*N* = 1003).

## Data Availability

The datasets used and/or analyzed during the current study are available from the corresponding author on reasonable request.
