# Supplementary material for: Behavioral and psychosocial factors and their effects on insomnia among people undergoing entry quarantine in hotels during COVID-19 pandemic: a cross-sectional study in Guangzhou, China
Source: BMC Public Health. 2023 May 30;23:996. doi: 10.1186/s12889-023-15340-4 (PMC10226877; doi:10.1186/s12889-023-15340-4)
Supplement: Supplementary file 1 — Additional file 1: Stable 1. Specific items of psychosocial scales (N = 1003). [file 12889_2023_15340_MOESM1_ESM.docx]

Stable 1. Specific items of psychosocial scales (*N* = 1003)

| Variable | N (%) |
| --- | --- |
| **Collectivism** |  |
| Item 1: Individuals should sacrifice their own interests for the collective |  |
| Strongly disagree and disagree | 65 (6.5) |
| Neutral | 229 (22.8) |
| Strongly agree and agree | 709 (70.7) |
| Item 2: Individuals should stick with the collective, even amid. hardship |  |
| Strongly disagree and disagree | 28 (2.8) |
| Neutral | 162 (16.1) |
| Strongly agree and agree | 813 (81.1) |
| Item 3: The welfare of the collective is more important than. the rewards of the individual |  |
| Strongly disagree and disagree | 48 (4.8) |
| Neutral | 208 (20.8) |
| Strongly agree and agree | 747 (74.4) |
| Item 4: The success of the collective is more important than. the success of the individual |  |
| Strongly disagree and disagree | 38 (3.8) |
| Neutral | 188 (18.7) |
| Strongly agree and agree | 777 (77.5) |
| Item 5: Individuals should pursue their own goals only after considering the welfare of the collective |  |
| Strongly disagree and disagree | 76 (7.6) |
| Neutral | 257 (25.6) |
| Strongly agree and agree | 670 (66.8) |
| Item 6: Loyalty to the group should be encouraged, even when. it undermines individual goals |  |
| Strongly disagree and disagree | 85 (8.5) |
| Neutral | 262 (26.1) |
| Strongly agree and agree | 656 (65.4) |
| **Social responsibility** |  |
| Item 1: Everyone should spend some time making their city or country better |  |
| Strongly disagree and disagree | 5 (0.5) |
| Neutral | 72 (7.2) |
| Strongly agree and agree | 926 (92.3) |
| Item 2: It is everyone’s duty to do their best to get the job done |  |
| Strongly disagree and disagree | 5 (0.5) |
| Neutral | 49 (4.9) |
| Strongly agree and agree | 949 (94.6) |
| Item 3: You feel bad when you cannot do the work you. promised to do |  |
| Strongly disagree and disagree | 61 (6.1) |
| Neutral | 172 (17.1) |
| Strongly agree and agree | 770 (76.8) |
| **Perceived people orientation of public health service** | 12.6±2.4 |
| Item 1: Public health service workers cared about my feelings and emotions |  |
| Strongly disagree and disagree | 46 (4.6) |
| Neutral | 163 (16.3) |
| Strongly agree and agree | 794 (79.2) |
| Item 2: I thought the public health service workers were trustworthy |  |
| Strongly disagree and disagree | 28 (2.8) |
| Neutral | 97 (9.7) |
| Strongly agree and agree | 878 (87.5) |
| Item 3: Public health service workers answered my questions in a way that I could understand |  |
| Strongly disagree and disagree | 31 (3.1) |
| Neutral | 114 (11.4) |
| Strongly agree and agree | 858 (85.5) |
